# Catarrhal Laryngitis

**Published:** 1900-06

**Authors:** Joseph Holt

**Affiliations:** New Orleans


### Catarrhal Laryngitis.

℞ Chloralis ....................gr. lxxv.
Potassii bromidi...............gr. xlv.
Ammonii bromidi.............gr. xxx.
Aquæ cinnamomi................oz. ij.

M. Sig.: Teaspoonful, and repeat in twenty minutes if not relieved. This is for a child of five years of age.

—*Joseph Holt, of New Orleans.*

